# Memristive neural network for on-line learning and tracking with brain-inspired spike timing dependent plasticity

**DOI:** 10.1038/s41598-017-05480-0

**Published:** 2017-07-13

**Authors:** G. Pedretti, V. Milo, S. Ambrogio, R. Carboni, S. Bianchi, A. Calderoni, N. Ramaswamy, A. S. Spinelli, D. Ielmini

**Affiliations:** 1 Dipartimento di Elettronica, Informazione e Bioingegneria, Politecnico di Milano and IU.NET, Piazza L. da Vinci 32, 20133 Milano, Italy; 2 Micron Technology, Inc., Boise, ID 83707 USA

## Abstract

Brain-inspired computation can revolutionize information technology by introducing machines capable of recognizing patterns (images, speech, video) and interacting with the external world in a cognitive, humanlike way. Achieving this goal requires first to gain a detailed understanding of the brain operation, and second to identify a scalable microelectronic technology capable of reproducing some of the inherent functions of the human brain, such as the high synaptic connectivity (~10^4^) and the peculiar time-dependent synaptic plasticity. Here we demonstrate unsupervised learning and tracking in a spiking neural network with memristive synapses, where synaptic weights are updated via brain-inspired spike timing dependent plasticity (STDP). The synaptic conductance is updated by the local time-dependent superposition of pre- and post-synaptic spikes within a hybrid one-transistor/one-resistor (1T1R) memristive synapse. Only 2 synaptic states, namely the low resistance state (LRS) and the high resistance state (HRS), are sufficient to learn and recognize patterns. Unsupervised learning of a static pattern and tracking of a dynamic pattern of up to 4 × 4 pixels are demonstrated, paving the way for intelligent hardware technology with up-scaled memristive neural networks.

## Introduction

Artificial intelligence, namely the ability to reproduce brain-like reasoning in a silicon chip, has been the objective of scientific research for the last 60 years^1^. Computers able to learn by sensory excitement from the external world, to infer abstract concepts and to make decisions, will spur the next technology revolution reshaping all aspects of our life and society. Recently, neural networks empowered with deep learning algorithms have shown the capability of playing games^2,3^, providing accurate translation of sentences^4^, and passing visual Turing tests^5^. These achievements were all demonstrated via software implementations in high-performance digital computers with conventional complementary metal-oxide-semiconductor (CMOS) technology. However, upscaling of these software approaches is frustrated by the von Neumann architecture of conventional computing machines where the processor and memory units are physically separate, thus resulting in large area, long time latency, and multichip system complexity. Also, there are fundamental power-density constraints affecting Moore’s law in the medium-long term which prevent future scaling of von Neumann computers to the complexity level required to emulate the brain^6^. Increasing research efforts are thus being directed at developing neural-network accelerators with suitable parallelism, low-power consumption and non-von Neumann, computing-in-memory architecture, suitable for performing brain-like tasks. For instance, a CMOS-based neuromorphic multi-core processor with one million neurons and 256 million synapses showed a reduction of power consumption by a factor 10^4^ with respect to the conventional CMOS architecture^7^. Low-power operation was also demonstrated in analog circuits with leaky integrate-and-fire (LIF) neurons and silicon synapses capable of spike-based visual pattern learning^8^ and solving complex constraint-satisfaction problems^9^. All these neuromorphic implementations rely on silicon CMOS synapses which are inherently volatile, binary, and poorly scalable. In fact, a CMOS-based static random access memory (SRAM) occupies a relatively large area of more than 100 F^2^, where F is the lithographic feature to manufacture the technology^10^. The logic state in the SRAM can be either 0 or 1, and is immediately lost upon turning off the power supply. A truly bio-realistic technology for neuromorphic systems requires a change of paradigm toward nonvolatile, multilevel, and scalable synapses consistent with the ultra-high density of connections (about 10^4^ synapses per neuron on average) in the human cortex^11^. In addition, the artificial synapses should display brain-inspired time-dependent weight update, such as spike-timing dependent plasticity (STDP)^12,13^, which is an essential feature of event-driven learning in biological neural networks.

Resistive/memristive devices, where the resistance changes in response to the application of an electrical stimulus, represent an ideal solution for electronic synapses in future neuromorphic systems^14,15^. At least 3 main categories of memristive devices have been described with reference to synaptic applications, namely resistive switching memory (RRAM) devices^16^, phase change memory (PCM) devices^17^, and magneto-resistive memory (MRAM) devices^18^. All types of memristive devices share the multilevel capability of changing their conductance to any arbitrary value within a possible range. The conductance is dictated by a nanoscale material modification, *e.g*., a structural phase distribution in PCM^19^, or a magnetic domain orientation in MRAM^20^, thus the multivalued conductance state can be retained even without any power supply. In addition, memristive devices show outstanding area efficiency thanks to their 2-terminal structure, which allows a minimum device size in the range of only few square-nm^21^, and stacking capability thanks to 3D integration^22,23^. Due to these beneficial properties, memristive devices have attracted strong interest as artificial electronic synapses in the last decade. In particular, the ability to update the synaptic weight by STDP has been verified in stand-alone synapses, such as RRAM^24–26^ and PCM^27,28^. Visual pattern training and recognition have been demonstrated by simulations of neuromorphic networks with memristive synapses^28–31^. Neuromorphic circuits with memristive synaptic arrays were experimentally evaluated by using recurrent Hopfield networks^32–34^ and perceptron networks, showing pattern classification^35^ and supervised weight-update via backpropagation^36^ or winner-take-all algorithms^37^. Bio-inspired unsupervised learning was only demonstrated in simulations^31^ or with a mixed set of hardware and software synapses^38^. All attempts were aimed at learning static patterns of a limited amount of pixels, although time evolution is an essential character of sensory information and enables object tracking in brain-inspired machine vision^39,40^. In this work, we demonstrate unsupervised learning of a static pattern and adaptation to a dynamic pattern within a perceptron-like network of memristive synapses where the weights are updated via local STDP^26,28,31^. Functional networks with up to 2 post-synaptic neurons are shown, supporting parallel neuromorphic computing and enabling future vision machines such as artificial retinas.

## Results

### Synaptic STDP characteristics

**Figure 1:**
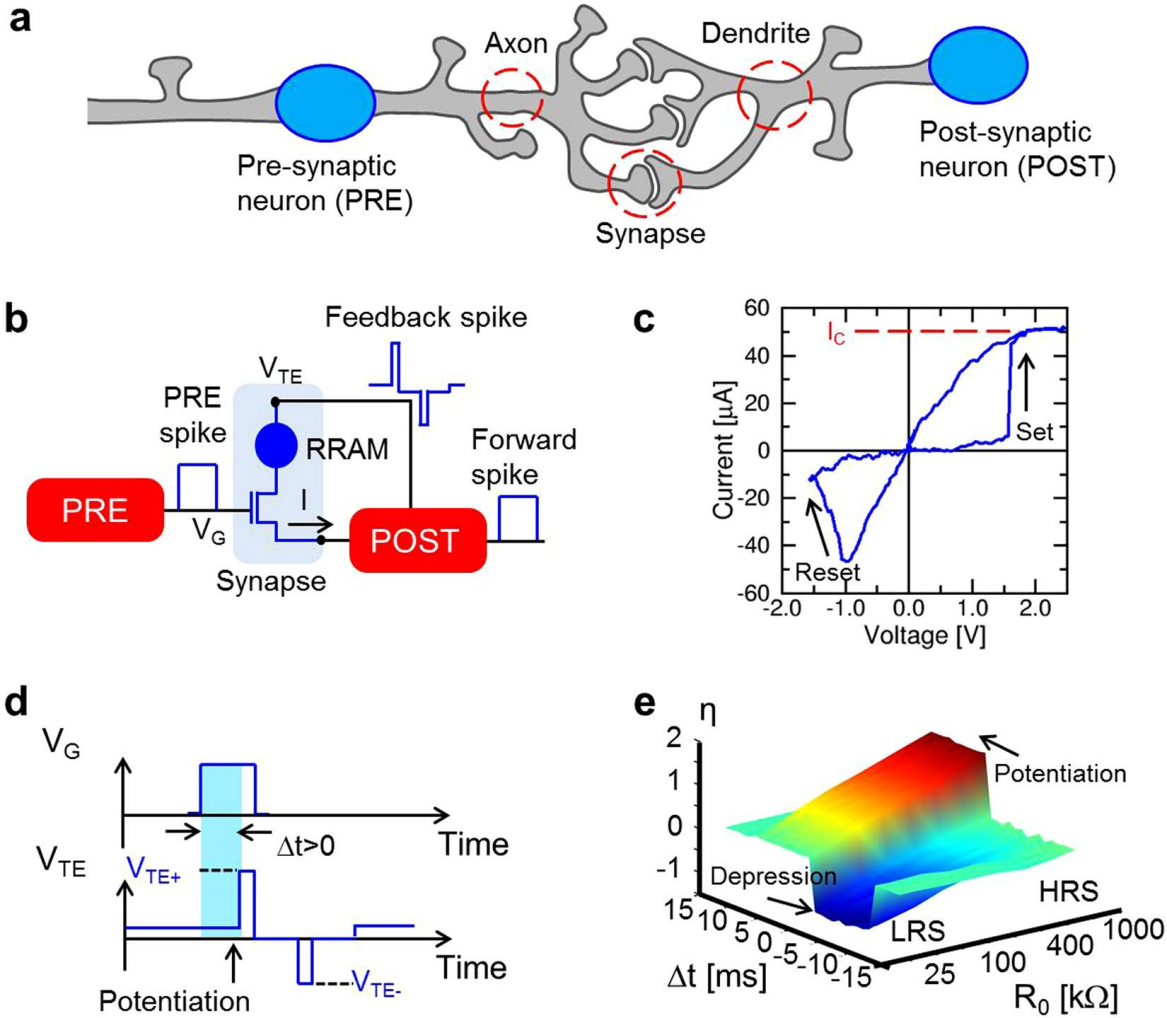
Synaptic device and characteristics. (**a**) Schematic structure of biological PRE, POST and synaptic connection between axon terminal and dendrite. (**b**) Schematic structure of the hardware PRE-synapse-POST unit: a PRE controls the FET gate of a 1T1R RRAM, while the POST receives the input current from the synaptic source and controls the synapse top electrode for inducing the synaptic current and stimulating potentiation/depression during the fire. (**c**) Measured I–V curve for a 1T1R RRAM synapse, showing set and reset transitions at positive and negative voltages, respectively, due to the bipolar operation of the HfO_2_ RRAM. (**d**) Schematic voltage traces of the PRE spike (top) and POST fire (bottom) signals for the case of potentiation (0 < Δt < 10 ms). (**e**) Experimental STDP characteristics, namely the measured conductance change η = R_0_/R as a function of the time delay between the PRE and POST spikes, for various initial synapse resistance values R_0_. Data indicate depression (η < 1) for −10 ms < Δt < 0 and potentiation (η > 1) for 0 < Δt < 10 ms. Initial LRS involves only depression, while initial HRS shows only potentiation.

Figure 1a shows the individual building block at the basis of any neural network, namely a synapse connected to a pre-synaptic neuron (PRE) and a post-synaptic neuron (POST). The synapse is responsible for the learning function in a neural network, since the synapse weight dictates the amount of signal that effectively reaches the POST upon PRE spiking. In our artificial neural network, the POST is represented by a LIF circuit while the synapse consists of a hybrid one-transistor/one-resistor (1T1R) structure^26,28,31^, as illustrated in the conceptual scheme of Fig. 1b. In this artificial synapse, the resistor is a RRAM device with a 10-nm thick switching layer of HfO_2_ (ref.^41^ and Fig. S1 of the Supplementary Information). As shown in Fig. 1c, the application of a positive voltage causes a transition to the low resistance state (LRS), called *set* process, as a result of the formation of a conductive filament (CF) containing oxygen vacancies between the 2 electrodes. The field-effect transistor (FET) in the 1T1R allows to limit the maximum current to a compliance current I_C_ during the set transition, thus providing control of the CF conductivity and avoiding irreversible breakdown^42^. The application of a negative voltage causes the retraction of the CF and the consequent transition to the high resistance state (HRS), called *reset* process.

The 1T1R synapse allows spike communication and STDP as detailed in Fig. 1b: when a PRE spike is applied to the gate terminal of the transistor, a positive current flows into the input terminal of the POST due to a positive static voltage V_TE_ at the top electrode, and is then integrated by the integrating stage of the LIF neuron. The result of the current integration is stored as an internal potential V_int_ (Fig. S2): as V_int_ exceeds a certain threshold V_th_, the neuron generates a forward spike, delivered to the next neuron, and a feedback spike, consisting of a sequence of positive and negative pulses, which are back-propagated to the synapse to allow for STDP^43^. As illustrated in Fig. 1d, if the POST-spike event follows the PRE-spike event, *i.e*., if the spike delay Δt = t_POST_ − t_PRE_ is positive, then the transistor is enabled by the PRE spike during the positive spike of the POST, which results in a set transition, or *synaptic potentiation*. On the other hand, if Δt < 0, then the transistor is enabled during the negative spike of the POST, thus causing a reset transition, or *synaptic depression*. Synaptic potentiation and depression controlled by spike timing delay Δt result in STDP, which was experimentally demonstrated by applying independent voltage pulses to the transistor gate and the top electrode of the synapse of Fig. 1b with variable timing delay Δt. After the application of voltage spikes, the resistance R of the 1T1R synapse was measured, allowing to determine the conductance change η defined as the inverse ratio between R and the initial resistance R_0_, namely η = R_0_/R. Figure 1e shows the measured η as a function of Δt and of the initial RRAM state R_0_. Potentiation (η > 1) occurs for 0 < Δt < 10 ms, except for relatively low R_0_ which is comparable to the target LRS resistance dictated by the gate voltage amplitude V_G_. On the other hand, depression (η < 1) takes place at −10 ms < Δt < 0 except for relatively high R_0_ which is comparable to the HRS resistance dictated by the top-electrode voltage V_TE_ (ref.^31^). When the delay time is larger than the gate pulse width, namely for |Δt| > 10 ms in this experiment, there is no overlap between pulses, thus the RRAM conductance is left unchanged. The time- and state-dependent plasticity in Fig. 1e is consistent with multiplicative STDP that is at the basis of self-adaptation^44^. The STDP response of the 1T1R synapse was also simulated by a physics-based analytical model for RRAM, showing good agreement with the experimental characteristics of Fig. 1e (see Fig. S3) and further supporting the STDP functionality of the 1T1R artificial synapse.

### Pattern learning in a neural network

**Figure 2:**
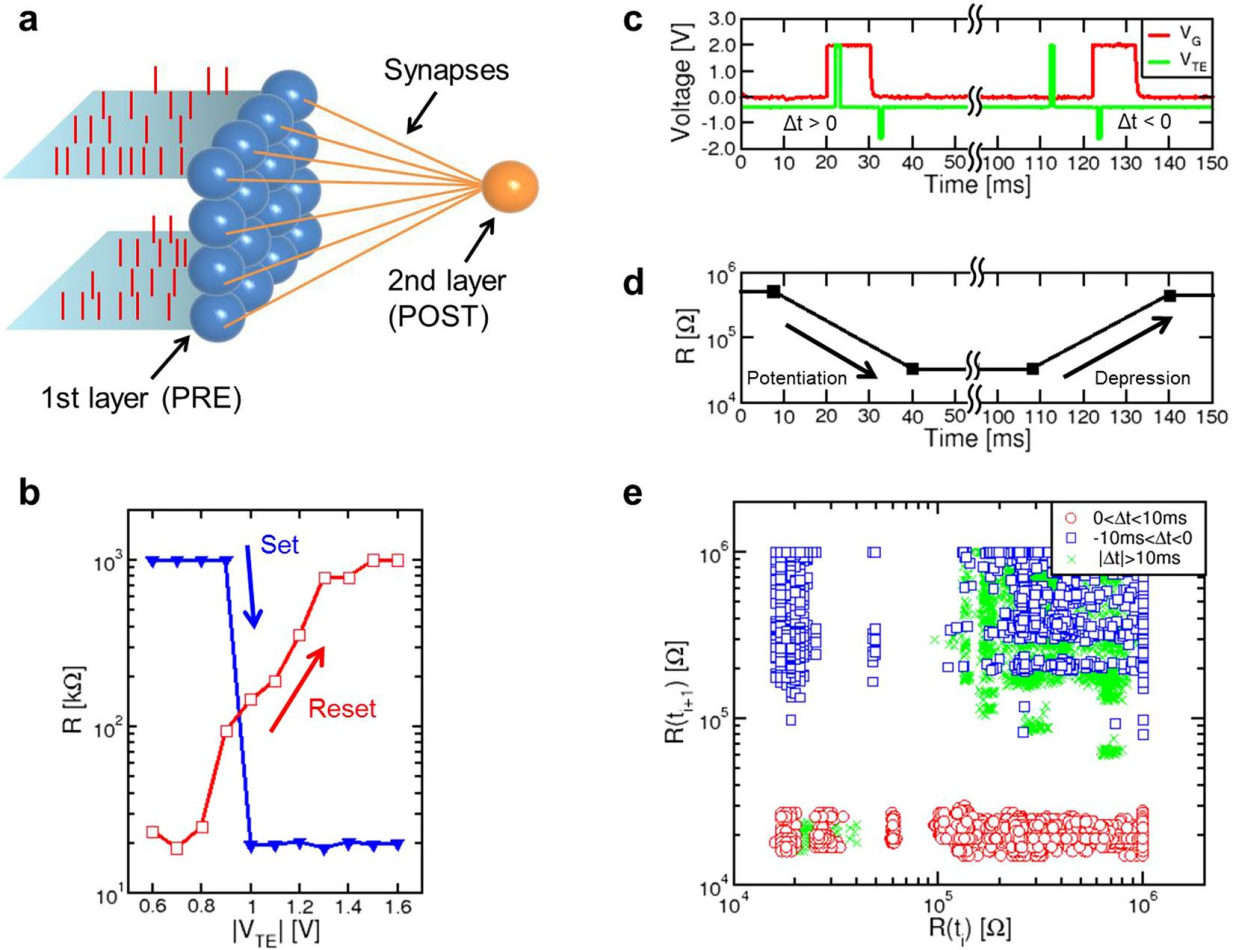
Synaptic network and operation. (**a**) Schematic illustration of the perceptron-like synaptic network including 16 PREs (first layer) and 1 POST (second layer), connected one to each other by 16 synapses. (**b**) Set/reset characteristics, namely synaptic R measured after the application of a 1 ms-long pulse, as a function of the pulse voltage. Set and reset characteristics were collected after preparing the synapse in the HRS and LRS, respectively. (**c**) Measured gate voltage V_G_ and top electrode voltage V_TE_, indicating the PRE spike and the POST backward spike, respectively, and (**d**) measured R before and after each pair of pulses, indicating potentiation for 0 < Δt < 10 ms and depression for −10 ms < Δt < 0. (**e**) Correlation plot showing the resistance R(t_i+1_) measured after the fire event as a function of R(t_i_) measured before the fire event. Potentiation, depression and no change are evidenced for 0 < Δt < 10 ms, −10 ms < Δt < 0, and |Δt| > 10 ms, respectively.

Learning of a visual pattern was experimentally demonstrated using the 2-layer perceptron network in Fig. 2a. The perceptron includes a first layer of 4 × 4 = 16 PREs, representing simplified retina neurons spiking in response to visual stimuli, and a single POST responsible for recognition and classification. Each PRE is connected to the POST by an artificial hybrid synapse capable of STDP. The neural network is operated in 2 phases: the first phase consists of *training* the network by stochastically submitting a visual pattern to the PREs to induce proper synaptic potentiation/depression by STDP, while the second phase consists of the *recognition* of patterns, where various patterns are submitted to the network to test the quality of learning. Learning is considered successful if the POST fires only in response to the same pattern used during training, whereas other patterns do not induce any fire, *i.e*., there are no false positives. Training relies on STDP occurring at any individual synapse in response to PRE stimulation and consequent POST fire events. STDP is usually disabled during recognition to avoid unwanted learning of false patterns.

The perceptron network was physically implemented by connecting PRE/POST neurons and synapses on a printed circuit board (PCB, see Fig. S4). To find the most appropriate voltages of the POST spike to induce potentiation or depression, pulses with increasing voltage and 1 ms width were applied and the resulting resistance change was collected. Figure 2b shows the measured R as a function of the absolute value of the pulse voltage |V_TE_|, indicating that the RRAM synapse completes the transition from high to low resistance at V_TE_ = 1 V, and from low to high resistance at V_TE_ = −1.5 V. In view of these set/reset characteristics and to take into account possible fluctuations of the set voltage V_set_ due to statistical variations of HRS^45^, the POST spike included a positive pulse of 2 V and a negative pulse of −1.6 V. Figure 2c shows examples of PRE and POST voltage spikes with a positive delay Δt = 3 ms, causing synaptic potentiation (Fig. 2d), followed by another pair of PRE and POST spikes with a negative delay Δt = −7 ms, causing synaptic depression. Figure 2e summarizes the effects of STDP by showing the correlation of resistance R(t_i+1_) measured after a spike as a function of R(t_i_) before the spike for cases of potentiation (0 < Δt < 10 ms), depression (−10 ms < Δt < 0), and no overlap between the PRE and POST spikes (|Δt| > 10 ms). The resistance decreases [R(t_i+1_) < R(t_i_)] for potentiation events and increases [R(t_i+1_) > R(t_i_)] for depression events, while all other cases show no change in the synaptic resistance [R(t_i+1_) ≈ R(t_i_)]. Note that the resistance after a single STDP event is either equal to the LRS or the HRS level, thus evidencing binary set/reset operations in the STDP characteristics.

**Figure 3:**
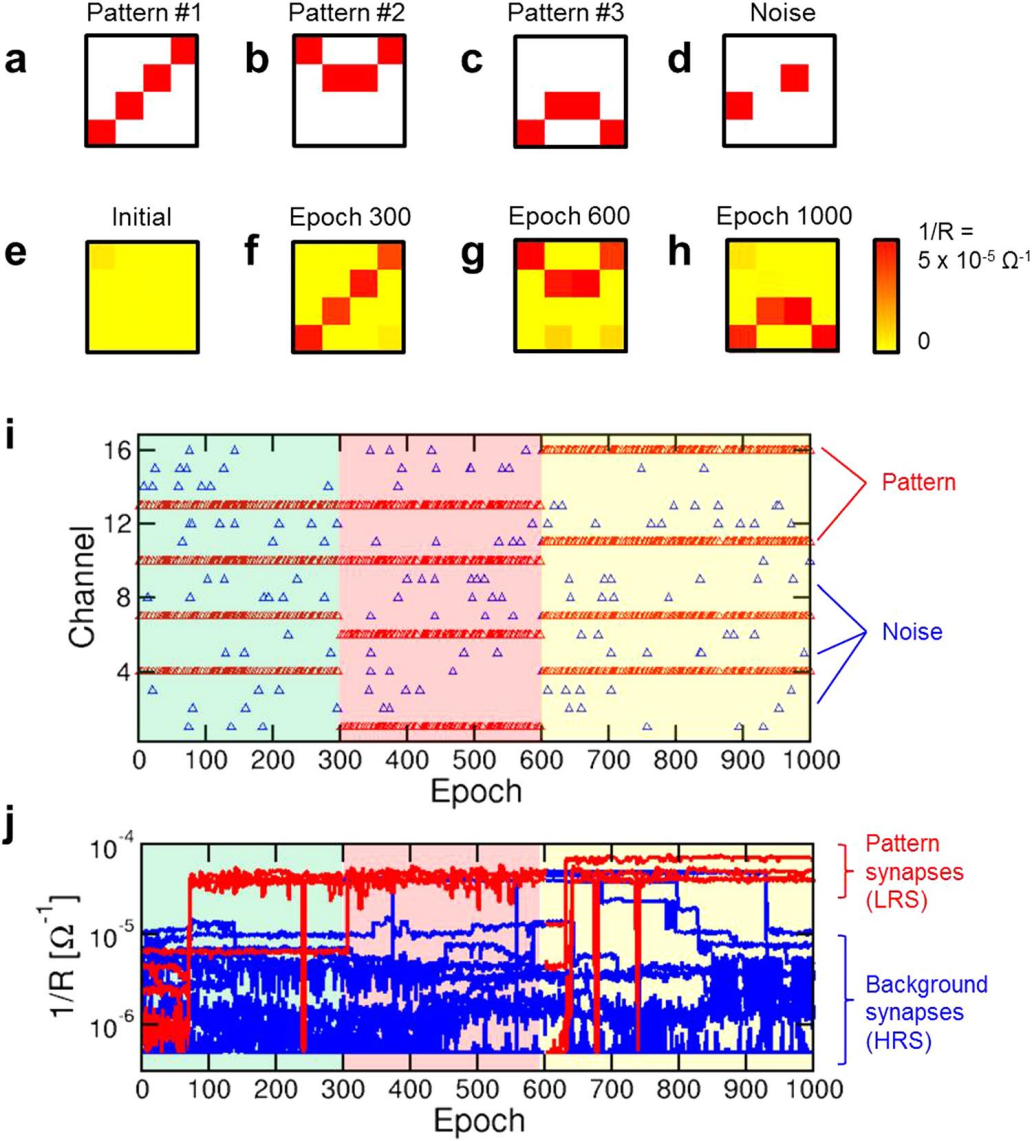
Static and dynamic learning within a 1-POST network. (**a,b,c,d**) Illustration of the pattern #1, pattern #2, pattern #3 and a typical random noise image that were submitted to the PREs during learning. (**e**) Initial configuration of synaptic weights, where all RRAM devices were prepared in HRS. (**f**) Configuration of synaptic weights after 300 epochs (3 s), indicating adherence to pattern #1 that was stochastically submitted during the first 300 epochs. (**g**) Configuration of synaptic weights after 600 epochs (6 s), indicating adherence to pattern #2 that was stochastically submitted during the previous 300 epochs. (**h**) Configuration of synaptic weights after 1000 epochs (10 s), indicating adherence to pattern #3 that was stochastically submitted during the previous 400 epochs. (**i**) Address of the PRE stimulation as a function of time, indicating the submission of the 3 patterns alternated with random noise. (**j**) Measured synaptic weights 1/R as a function of time: pattern weights (red) and background weights (blue) tend to high and low conductance, respectively, thus demonstrating pattern learning.

After verifying the STDP at the level of single synapse, we tested learning of predefined images of 4 × 4 pixels. The synaptic network was first trained with a first image, namely the diagonal pattern #1 in Fig. 3a, to test the learning of a static image, then patterns #2 (Fig. 3b) and #3 (Fig. 3c) are subsequently submitted to demonstrate dynamic learning. A stochastic training approach was adopted, where PRE spikes alternatively present the image or a random pattern (*e.g*., see Fig. 3d), consisting of only 3% of the pixels on average being randomly activated^31^. Image and noise were alternatively submitted at each epoch, consisting of an individual time fragment of 10 ms width. The probabilities of presenting pattern and noise were equally set to 50%. The synaptic weights were initially prepared in a high resistance state, as indicated in Fig. 3e. The threshold voltage was set to V_th_ = 0.72 V.

During the first 300 epochs of training with pattern #1, the image was readily learnt because of STDP causing potentiation of image synapses and depression of background pixels. Static learning of pattern #1 is evidenced in Fig. 3f, where all pattern synapses show LRS conductance, while background synapses show HRS conductance. As the submitted image is changed from pattern #1 to pattern #2, the learning of pattern #2 is demonstrated, as evidenced by the final synaptic weights after 600 epochs in Fig. 3g. This supports ‘dynamic’ learning, or adaptation of synaptic weights to the presented image in real time by our neuromorphic system. Similarly, pattern #3 is learnt during the third training phase between epoch 600 and epoch 1000, as shown by the final synaptic weights in Fig. 3h. Figure 3i shows the PRE spikes as a function of epochs, indicating the 3 sequential training phases, while Fig. 3j shows the corresponding time evolution of synaptic weights 1/R for synapses stimulated by the pattern, or simply *pattern synapses* in the following (red), and synapses located outside the pattern, referred to as *background synapses* in the following (blue). A movie showing the evolution of the synaptic weights in a color plot similar to Fig. 3a–h is available in the Supplementary Movie 1.

Pattern and background weights show STDP-induced potentiation and depression, respectively, in each of the 3 training stages. Selective synapse potentiation/depression can be understood as follows: as the pattern is submitted at epoch *i*, V_int_ increases significantly, thus potentially inducing a fire event. This causes STDP with Δt > 0, hence potentiation (Fig. 2c–e). On the other hand, if a noise pattern is submitted at epoch *i* + 1 after fire, then STDP with Δt < 0 takes place, thus causing depression of the corresponding synapses. As a result, selective potentiation takes place at pattern synapses, while unselective depression takes place throughout the whole synaptic network. By properly adjusting the noise percentage in a range between 2% and 7% of randomly activated pixels, stable learning can be achieved. A larger percentage of noise would cause fire in response to the submission of noise, which induces potentiation of random synapses and depression of pattern synapses, thus is to be avoided. Static learning similar to the one in the first 300 epochs in Fig. 3 can be demonstrated irrespective of the initial configuration of synaptic weights, which can be prepared in either HRS (Fig. 3), LRS (Fig. S5), or random states (Fig. S6). The independence on the initial configuration of weights is due to the STDP inducing selective potentiation and unselective depression of synapses, and is essential for the dynamic learning in Fig. 3, where a new image must overwrite the previous one by potentiating weak synapses and depressing strong synapses where needed.

While neuromorphic systems are generally expected to operate in the same timescale (10 to 100 ms) as the biological counterparts, *e.g*., to enable gesture and speech recognition, our RRAM devices are also capable of much faster learning and recognition via STDP. High-speed learning can be achieved by using the same hardware operated with 100 times shorter pulses, *i.e*., PRE spikes of 100 μs width and POST positive/negative pulses of 10 μs width (Fig. S7). A higher feedback voltage V_TE+_  = 3.3 V was used to enable set transition in the 10 μs timescale. Given the proportionality between energy and time, accelerated STDP can also be used to reduce energy consumption during learning^28^. Time flexibility of RRAM devices thus allows to match various time/energy requirements depending on the specific application scenario.

STDP in our approach is implemented as a deterministic binary plasticity rule, i.e., positive delay results in full set transition to the LRS, while negative spike delay causes full reset transition to the HRS. This is also dictated by the binary switching characteristics of our device in Fig. 1c, where both set and reset transitions appear quite abruptly as the voltage exceeds the set or reset threshold. However, for certain applications, analog weight variation may be useful, e.g., vision does not only imply recognition of object shapes, but also textures and colors, which can be represented by analog weights. Analog STDP with inherently digital RRAM devices was previously obtained by probabilistic potentiation/depression, where application of a voltage close to the threshold results in set/reset only in a random subset of cases^46^. Here, we adopted a different approach to achieve analog weight potentiation. To demonstrate learning of gray-scale images, we represented different gray tones through variable PRE spike voltage amplitudes (Fig. S8 and Supplementary Movie 2). An increasing value of V_G_ results in an increasing transistor current I_C_ during the set operation, which controls the LRS resistance^25,26^. As a result, synapses stimulated by a light gray intensity (high V_G_) are potentiated to a high conductance, while a dark gray intensity yields low conductance. Similarly, color-scale images can be represented by multiple synapses per pixel where each synapse represents the intensity of a color component, *e.g*., adopting a RGB representation^43^.

### Image recognition

**Figure 4:**
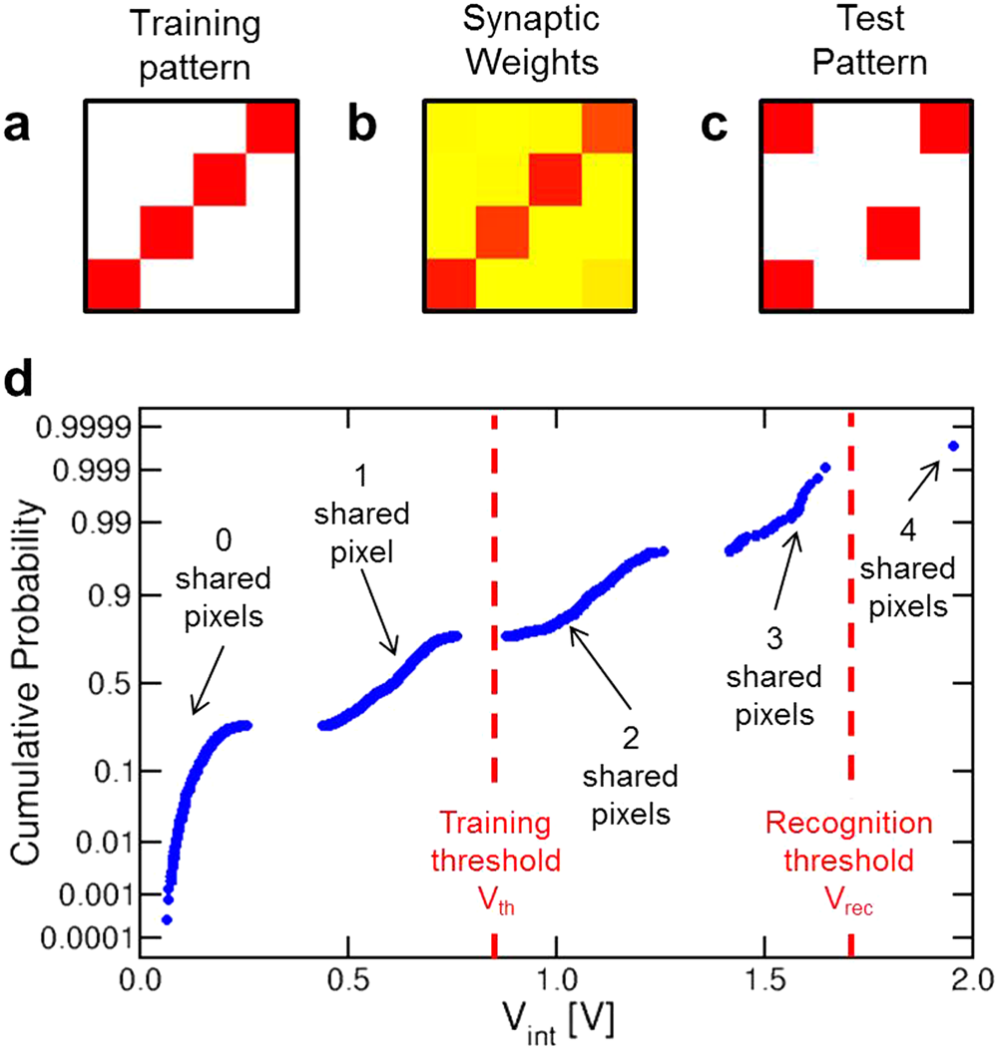
Pattern recognition. (**a,b,c**) The pattern that was submitted during the training phase, the corresponding synaptic weights after 300 training epochs, and one of the possible 1820 test patterns that were submitted during the recognition phase. (**d**) Cumulative distribution of the internal potential values measured after the presentation of the recognition patterns. Each sub-distribution corresponds to a given number of pixels shared with the training pattern. By adopting a threshold voltage V_rec_ = 1.7 V during recognition, only one pattern, *i.e*., the training pattern, could trigger fire in the POST, thus preventing any false positive.

The second key function of a perceptron network is the pattern recognition, that is the capability to discriminate between patterns that were previously submitted during the learning phase. In the recognition phase, an image is presented to the network while monitoring the response of the POST. The POST should fire in response to an image which is similar (or perfectly equivalent) to the one submitted during training, *i.e*., the *training pattern*. In addition, recognition should result in no false positives, namely, the POST should not fire in response to patterns which are significantly different from the training pattern. To test the recognition capability, we statically trained our network with the training pattern of Fig. 4a, resulting in the final synaptic weights of Fig. 4b after 300 epochs. Then we submitted a sequence of all 1820 *test patterns* (see, *e.g*., Fig. 4c) with 4 activated pixels out of 16, *i.e*., the same number of activated pixels as in the training pattern. After submitting any test pattern, we checked for a possible POST fire event and discharged the internal potential V_int_ for a new test. Figure 4d shows the cumulative distribution of the 1820 calculated values of V_int_, obtained after integrating the total current I_post_ given by:

1 $${I}_{post}={V}_{TE}\sum _{n=1}^{16}{R}_{n}^{-1}$$

where R_n_ is the resistance of the *n*-th synapse. Note that the latter consists of a 1T1R structure, thus R_n_ includes both contributions from the transistor, which is conductive only for the activated pixels of the test pattern, and the memristor, which is conductive (LRS) only within the pattern which was submitted in the training phase. The distribution shows five sub-distributions, corresponding to patterns sharing no pixels with the training pattern (V_int_ ≈ 0), and patterns sharing 1, 2, 3 or 4 pixels with the training pattern, showing increasing values of V_int_. In this recognition experiments, the threshold voltage V_rec_ for fire was set to 1.7 V, which led to a fire event only in correspondence of the presentation of the training pattern, *i.e*., no false positives were recorded. These results support the pattern recognition capability of our synaptic network.

### Multiple pattern learning and tracking

**Figure 5:**
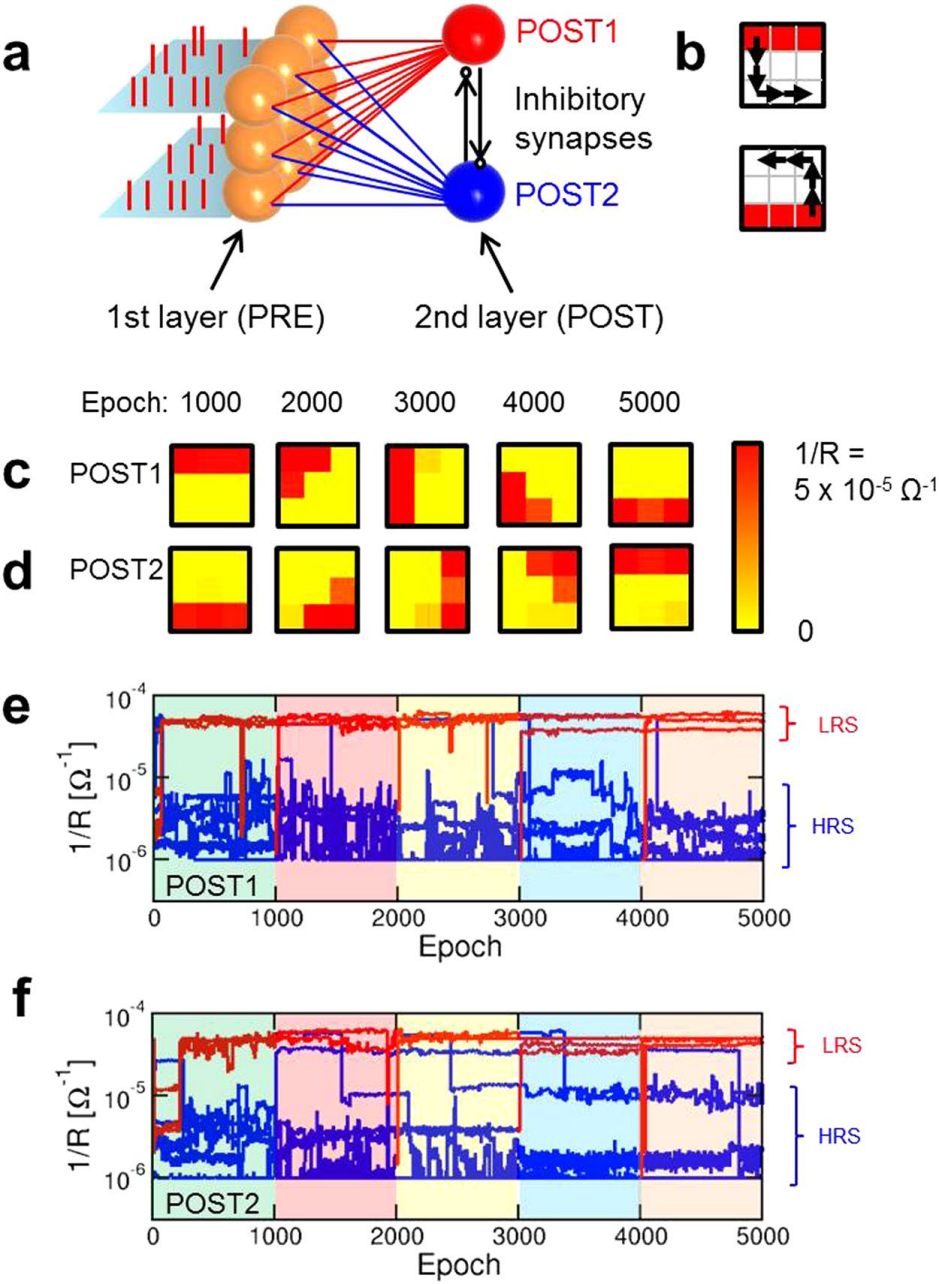
Static and dynamic learning within a 2-POST network. (**a**) Schematic illustration of a perceptron network with a 3 × 3 PRE layer and 2 POSTs, with 18 synapses between the PRE and POST layers. Inhibitory synapses connect the 2 POSTs to reduce the internal potential of one POST when the other POST fires. (**b**) Patterns submitted during the first phase (top and bottom bars for static learning) and sequence of 4 pattern shifts for the dynamic learning phase. (**c,d**) Synaptic weights for POST1 and POST2 at the end of each sequence of learning indicated in (**b**). (**e,f**) Time evolution of the synaptic weights 1/R for POST1 and POST2 during the 5 phases of the dynamic learning. Pattern weights (red) and background weights (blue) tend to LRS and HRS, respectively.

Unsupervised learning in the brain usually proceeds by simultaneous specialization of distinct neurons in response to sensory stimuli^47^. To enable multiple image learning, we extended our network to include one additional POST as shown in Fig. 5a. POST1 and POST2 are both fully connected by separate synapses to the first layer of PRE^31^. The operation of the 2-neuron network is the same as the 1-neuron network of Figs 1–4, except for the presence of lateral inhibitory synapses between the 2 POSTs. When POST1 fires, a spike is sent through the inhibitory synapse to POST2 to reduce its internal potential V_int,2_ by a fixed amount (40% in our experiment). Similarly, when POST2 fires, a spike through the inhibitory synapse to POST1 forces V_int,1_ to decrease by the same amount. This winner-take-all approach prevents the 2 neurons to specialize to the same image, thus allowing the maximization of the network learning and recognition functionalities^48^. Complex neuron networks with inhibitory synapses have also been shown to enable parallel computing tasks, including tackling NP-hard to a certain level, Sudoku games and similar constraint satisfaction problems^49^.

We first tested static training in the 2-neuron network by submitting the 2 images of 3 × 3 size in Fig. 5b. Static training was continued for 1000 epochs using the usual stochastic approach with alternated patterns (Fig. 5b) and random noise. After the initial training, the 2 images were shifted counter-clockwise along the perimeter of the 3 × 3 square as indicated by arrows in Fig. 5b. Images were moved by a total of 4 steps, and after each step the image was submitted for 1000 epochs to verify the ability of our network to track the moving image. Results are shown in Fig. 5c for POST1 and Fig. 5d for POST2, reporting the final synaptic weights at the end of each training phase. Not only the static learning of patterns in Fig. 5b is demonstrated by the 2 neurons after 1000 epochs, but  also each modified pattern is correctly learnt at the end of each phase of the dynamic learning. Note that each neuron remains locked to one specific image during its movement, since this minimizes the number of synapses (2 for each POST) that must change their weights. The synaptic weights 1/R are shown as a function of time in Fig. 5e for POST1 and Fig. 5f for POST2, while the Supplementary Movie 3 shows an overview of the time evolution of synaptic weights during learning and tracking of the moving image. The results confirm that synaptic weights can track dynamic patterns as a result of on- line unsupervised learning.

## Discussion

Our results support object learning, recognition and adaptation in synaptic networks by unsupervised Hebbian learning, which is believed to be a fundamental synaptic plasticity principle within the human brain. Hebb’s rule generally describes a reward scheme where neurons firing in a causal sequence are awarded with incremented synaptic connection, while neurons firing with apparently uncorrelated timing are penalized with a decremented synaptic connection^50^. In machine learning, unsupervised techniques find application in data clustering and anomaly detection, which is the standard methodology to monitor intrusion hazards, bank frauds, medical errors, and similar threats^51^. In biological systems, reward schemes have been evidenced in several sensory functions such as vision^52^, olfactory system^53^, and sensory-motor system^54,55^. Even the ability to recognize and anticipate the direction of moving objects, which is fundamental for the control of autonomous robots and vehicles, has been modeled by burst-mode STDP in the visual cortex^56^. The ubiquitous character of STDP suggests that physical hardware capable of STDP might have a key role in the development of humanoid robots and other artificial systems aiming at mimicking human perception and cognition. Thanks to the bio-mimetic nature of STDP, unsupervised synaptic networks might enable neuro-prosthetics technologies, where implanted hardware interconnected with biological neurons can supply and complement various brain functionalities to correct disabilities and heal injuries. Similarly, hardware systems based on STDP or other bio-realistic plasticity rules might be designed to replicate, or at least imitate, certain areas of the human brain *in silico*, thus helping to understand human cognition and perception.

A key limitation to meet these challenges is the difficulty to understand and recreate the architecture of biological neural networks. For instance, the visual cortex is organized into 8–10 functional layers, with various types of neurons and complex arrangement of synaptic connections within the axon arbor^39,57^. Replication and unsupervised training of such deep networks with STDP and other spike time-dependent rules is not yet understood and achieved in hardware. In addition, the response in the neural network can be extremely complicated, including short-term and long-term plasticity, excitatory and inhibitory synaptic response, and various types of network-level behaviors, such as feedforward or recurrent spike propagation. Various forms of plasticity rules have been proposed, including not only STDP but also rate-based and triplet-based learning^58^. Recreating the deep architecture and complex phenomenology within hardware requires a detailed understanding of the structure and operation of the brain. In this scenario, our STDP synaptic memristive network offers a flexible building block to build up-scaled spiking networks to mimic learning and processing in the human brain.

In summary, we presented a neural network with memristive synapses capable of STDP. Stochastic learning relies on the alternated presentation of pattern images and random noise, to enable potentiation and depression, respectively. As a result, unsupervised learning of static and dynamic images, and recognition of the same patterns were demonstrated. The demonstrated concept might provide a fundamental building block for scalable, low-power, brain-inspired computing hardware based on memristive devices.

## Methods

### RRAM synapses

The RRAM devices used in this study consist of a 10-nm thick switching layer of HfO_2_ which was deposited by atomic layer deposition (ALD) on top of a lithographically-confined bottom electrode made of TiN. A cross-section TEM photograph of the device is shown in Fig. S1. The HfO_2_ layer was doped with silicon and deposited in the amorphous phase, as confirmed by diffraction studies^41^. A reactive Ti top electrode was deposited on top of the HfO_2_ dielectric layer, to act as oxygen scavenger, leading to oxygen exchange layer (OEL) of TiO_x_ between Ti and HfO_2_. The OEL was instrumental in increasing the concentration of oxygen vacancies in HfO_2_, thus enhancing the leakage current in the pristine state and reducing the forming voltage. Forming was operated by the application of 100 ms-long pulses of 3 V amplitude, to initiate the CF creation and the related resistive switching process by a controlled soft-breakdown of the dielectric layer. The RRAM was connected to a FET, which was integrated in the front-end of the same silicon chip by conventional complementary-metal-oxide-semiconductor (CMOS) process. The resulting 1T1R structure was controlled during forming, set, and reset by connecting its 3 terminals, namely the FET gate, the FET source and the top electrode of the RRAM. The dc conduction and bipolar switching characteristic of the RRAM (Fig. 1c) were collected by an HP4155B Semiconductor Parameter Analyzer connected to the experimental device within a conventional probe station for electrical characterization.

### Synaptic network

The 1T1R synapses were connected to an Arduino Due microcontroller (μC) on a PCB for experiments on the neural network. The PCB hosted up to 18 RRAM chips, each containing a 1T1R synapse, and all of them connected with their 3 terminals according to the schematic of Fig. S4. In the network, each PRE represented an axon terminal, controlled by the μC and connected to a synapse gate. All synaptic top electrodes were driven by the μC and normally biased to V_bias_ = −0.2 V to induce a small current through the 1T1R synapses under a PRE spike. All source terminals were connected to the POST input, consisting of a transimpedance amplifier (TIA), enabling current-to-voltage conversion. The output voltage of the TIA was fed into an input terminal of the μC’s ADC for digital integration to describe the first stage of the POST. The internal threshold potential was tuned to enable firing in correspondence of 2 PRE spikes activating full-LRS synapses. At the fire event, the voltage controlling the synaptic top electrodes was switched from V_bias_ to the V_TE+_ and V_TE−_ according to the pulse trace in Fig. 2c, to induce time-dependent potentiation or depression. To operate the network, the PRE spike sequence was first stored in the internal memory of the μC, then the sequence was launched while monitoring the synaptic weights 1/R and the internal potential V_int_ at each epoch. The spike and fire voltages and the input currents were also monitored by a Lecroy Waverunner oscilloscope with 600 MHz bandwidth and maximum 4 GSample/s sampling rate.

## Electronic supplementary material

Supplementary Information

Movie 1

Movie 2

Movie 3
