# Publisher Correction: Memristive neural network for on-line learning and tracking with brain-inspired spike timing dependent plasticity

**DOI:** 10.1038/s41598-018-26716-7

**Published:** 2018-06-18

**Authors:** G. Pedretti, V. Milo, S. Ambrogio, R. Carboni, S. Bianchi, A. Calderoni, N. Ramaswamy, A. S. Spinelli, D. Ielmini

**Affiliations:** 1 Dipartimento di Elettronica, Informazione e Bioingegneria, Politecnico di Milano and IU.NET, Piazza L. da Vinci 32, 20133 Milano, Italy; 2 Micron Technology, Inc., Boise, ID 83707 USA

Correction to: *Scientific Reports* 10.1038/s41598-017-05480-0, published online 13 July 2017

The original PDF version of this Article contained a truncated version of Figure 1, where Figure 1d and Figure 1e were missing.

Additionally, the Acknowledgements section was omitted initially from the Article and it now reads:

“This work was supported in part by the European Research Council (grant ERC-2014-CoG-648635-RESCUE).”

These errors have now been corrected in the HTML and PDF versions of this Article.

