# Supplementary material for: Memristive neural network for on-line learning and tracking with brain-inspired spike timing dependent plasticity
Source: Sci Rep. 2017 Jul 13;7:5288. doi: 10.1038/s41598-017-05480-0 (PMC5509735; doi:10.1038/s41598-017-05480-0)
Supplement: Supplementary file 1 — Supplementary Information [file 41598_2017_5480_MOESM1_ESM.pdf]

Supplementary Information

Memristive neural network for on-line learning and tracking with brain-inspired spike timing dependent plasticity

G. Pedretti[1], V. Milo[1], S. Ambrogio[1], R. Carboni[1], S. Bianchi[1], A. Calderoni[2], N. Ramaswamy[2], A. S. Spinelli[1], & D. Ielmini[1*]

[1]*Dipartimento di Elettronica, Informazione e Bioingegneria, Politecnico di Milano and IU.NET, Piazza L. da Vinci 32 – 20133 Milano, Italy*

[2]*Micron Technology, Inc., Boise, ID 83707 USA*

[*]Contact: daniele.ielmini@polimi.it

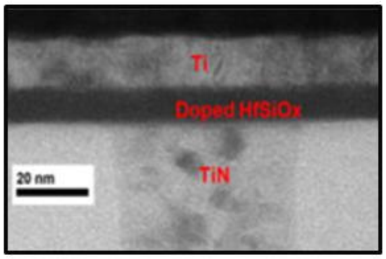

Figure S1 | TEM cross section of the synaptic memristor. Synapses in our work consisted of one-transistor/one-resistor (1T1R) integrated structures including a field-effect transistor (FET) and a resistive switching memory (RRAM) device. The TEM shows a cross section of the RRAM device stack[1], including a 10-nm thick switching layer of Si doped HfO_2 deposited by atomic layer deposition (ALD) on a confined TiN bottom electrode with 50-nm diameter. After deposition of the Ti top electrode, partial Ti oxidation led to depletion of oxygen from the HfO_2 layer, as revealed by XPS profiling[1], within an interfacial oxygen exchange layer (OEL). The OEL was functional in controlling leakage and forming in the pristine state of the RRAM.

Resistive switching in the RRAM device was initiated by forming, i.e., a soft breakdown operation by the application of a forming voltage of 3 V for 100 ms. Afterwards, set and reset transitions could be induced by the application of positive and negative voltages, respectively, in either DC or pulsed conditions. The integrated transistor in the 1T1R structure enabled the control of the maximum current during the set transition, thus allowing to maintain a top boundary of conductivity for the low resistance state (LRS) which is instrumental in achieving multiplicative spike timing dependent plasticity (STDP) and homeostatic plasticity.

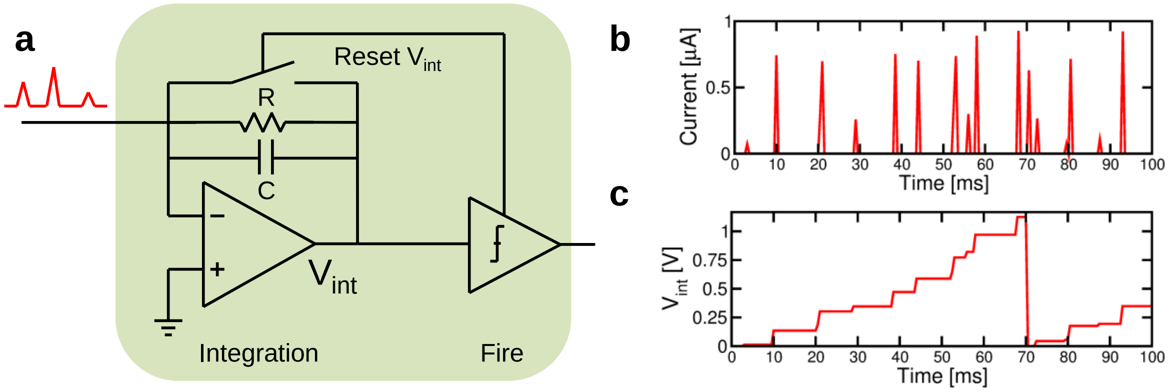

Figure S2 | LIF neuron. (a) Schematic illustration of a LIF neuron, (b) calculated random input spiking current, and (c) corresponding integrated signal V_{int}. In the LIF neuron, the first stage (integration) integrates the input spiking current, whereas the second stage (fire) delivers a spike pulse as the internal potential reaches a threshold voltage V_{th}. At fire, a forward spike is sent to the next-layer neuron, while a backward spike is sent to the synapse for STDP. A signal is sent to the first stage to reset V_{int} for the next integration ramp. The current in (b) was calculated assuming current spikes with both random amplitude and random timing according to a Poisson distribution of events.

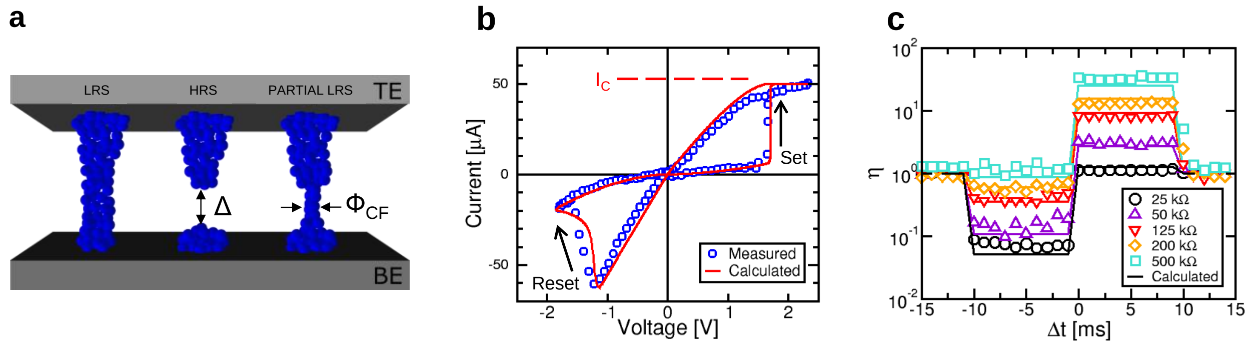

Figure S3 | Analytical model of the RRAM device. (a) Schematic illustration of the RRAM device model, showing the conductive filament (CF) shape in the LRS (left), in the high-resistance state (HRS, center) and in an intermediate state during set transition, also known as partial LRS (right). The CF consists of a region of the device with a large concentration of defects, such as metallic impurities and oxygen vacancies, which cause a local enhancement of conductivity due to a doping mechanism[2]. In the LRS, the CF is fully connected between the top electrode (TE) and the bottom electrode (BE). When a negative voltage is applied to the TE, defects migrate toward the TE, thus causing the depletion of the CF in the region close to the BE. The analytical model describes the gradual increase of the depleted region width Δ with time, according to the rate equation[3]:

$$\frac{d\Delta}{dt} = Ae^{-\frac{E_A}{kT_{reset}}} \quad \text{(S1)}$$

where A is a constant, E_A is the activation energy for defect migration, k is the Boltzmann constant and T_{reset} is the local temperature at the BE-side boundary of the depleted region. As Δ gradually increases during reset, the RRAM resistance increases because of the insulating gap. On the other hand, the application of a positive voltage to the TE results in a set transition, where the CF is recreated within the depleted gap due to ionic migration toward the BE. The model describes set transition by the rate equation:

$$\frac{d\phi_{CF}}{dt} = Ae^{-\frac{E_A}{kT_{set}}} \quad \text{(S2)}$$

where ϕ_{CF} is the diameter of the CF in the depleted region and T_{set} is the local temperature at the TE-side boundary of the depleted region. As ϕ_{CF} increases during set transition, the RRAM resistance decreases abruptly due to the recreation of the CF. (b) Measured and calculated I-V curves of a HfO_2 RRAM device, demonstrating the accuracy of the physics-based analytical model in capturing the shape and position of the set and reset transitions. (c) Measured and calculated $\eta = R_0/R$, namely the relative change of conductance after a pair of PRE/POST spikes in STDP, as a function of spike delay Δt, for various initial resistance R_0. Only potentiation is seen for high R_0, while only depression is seen for low R_0, due to the limited range of conductance of the RRAM synapse. The model allows to accurately predict the STDP behavior of the 1T1R synapse. Random fluctuations of R were assumed in the model (not shown in the figure) to capture the stochastic behavior of the RRAM synapse[4].

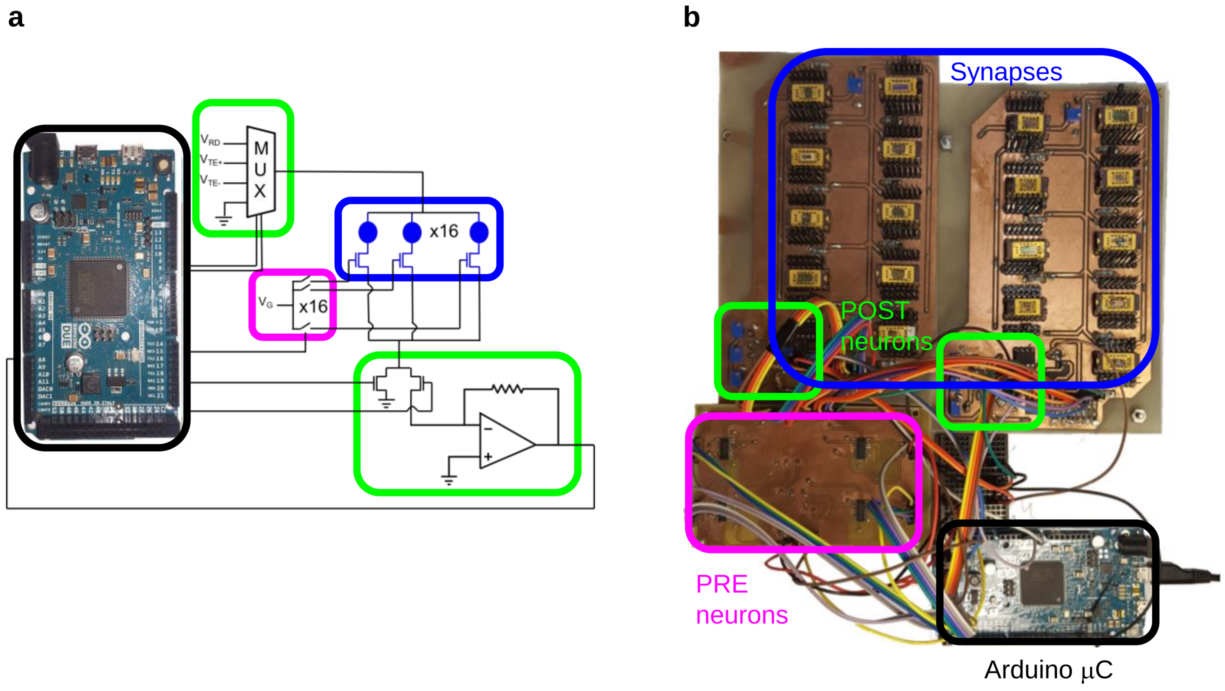

Figure S4 | Neural network for STDP learning. (a) Schematic illustration of the neural network circuit, including 1T1R synapses, PRE neurons, POST neurons, and an Arduino Due microcontroller (μC) for controlling the PRE/POST neurons. The POST circuit includes a trans-impedance amplifier (TIA) for converting the synaptic current into a voltage, and a multiplexer (MUX) dictating the top electrode voltage V_{TE}, which could change between V_{RD}, V_{TE+} and V_{TE-}. The PRE circuit consists of switches connecting the synaptic gates (which were normally grounded) to a voltage level V_G during PRE spikes. PRE and POST blocks were digitally controlled by the μC, which was connected to a computer by a serial bus for program upload and data download. (b) Picture of the printed circuit board (PCB) supporting the neural network. 16 synaptic chips were wire-bonded to a dual-in-line (DIL) chip holder for the experimental demonstration of learning with 4x4 PREs and one POST.

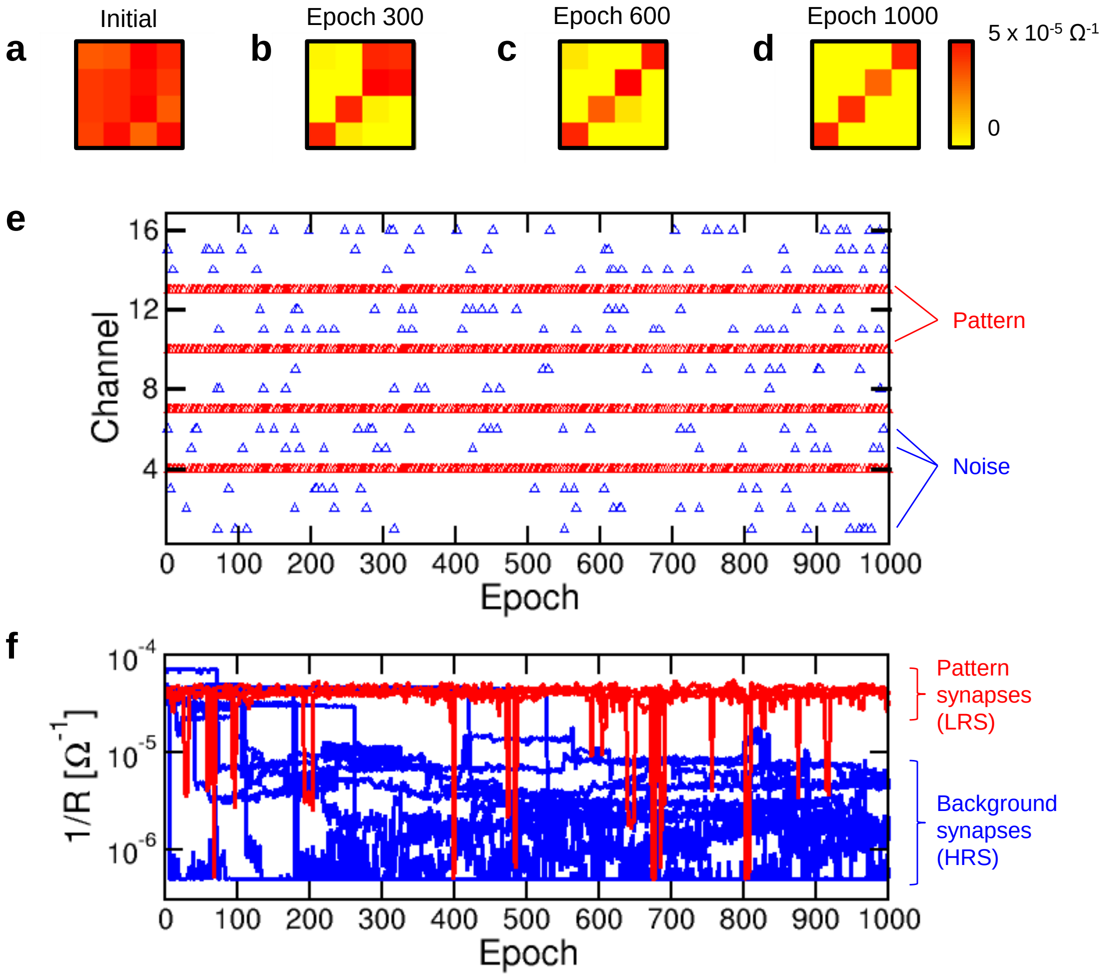

Figure S5 | Pattern learning with synapses initially prepared in LRS. (a,b,c,d) Color code representation of the synaptic conductance at time 0, after 300 epochs, after 600 epochs, and after 1000 epochs, respectively. The color scale goes from yellow (HRS) to red (LRS). A diagonal pattern stochastically alternated with random noise was presented. (e) Sequence of the PRE spikes, showing the active PRE channels at each epoch during the experiment. Red and blue symbols indicate active PRE channels during the presentation of the pattern (4 diagonal pixels) and during the presentation of noise, respectively. (f) Synaptic weights 1/R as a function of time during the experiment. Red and blue lines correspond to synaptic weights in the pattern and in the background, respectively. Background synapses are initially in LRS and tend to HRS as a result of STDP depression.

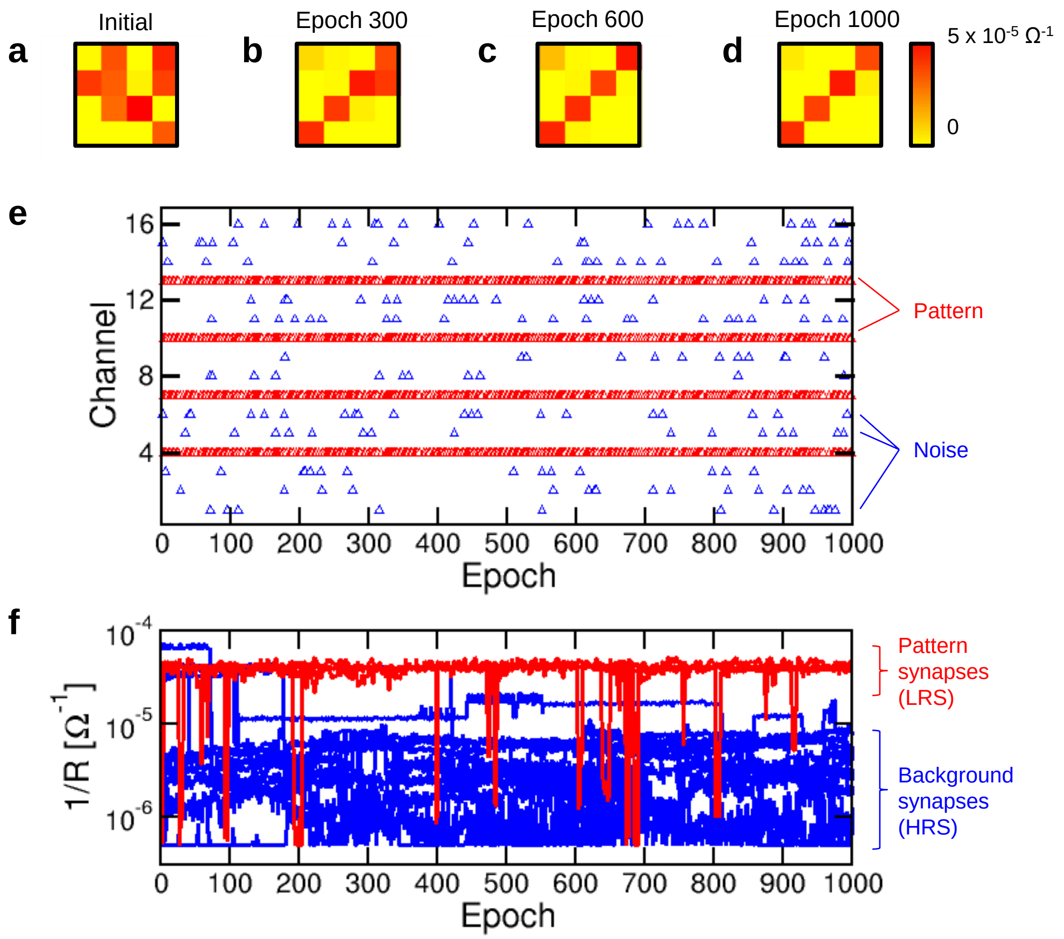

Figure S6 | Pattern learning with synapses initially prepared in random states. (a,b,c,d) Color code representation of the synaptic conductance at time 0, after 300 epochs, after 600 epochs, and after 1000 epochs, respectively. The color scale goes from yellow (HRS) to red (LRS). A diagonal pattern stochastically alternated with random noise was presented. (e) Sequence of the PRE spikes (same as in Fig. S5), showing the active PRE channels at each epoch during the experiment. Red and blue symbols indicate active PRE channels during the presentation of the pattern (4 diagonal pixels) and during the presentation of noise, respectively. (f) Synaptic weights 1/R as a function of time during the experiment. Red and blue correspond to synaptic weights in the pattern and in the background, respectively. Pattern synapses tend to LRS while background synapses tend to HRS, irrespective of their initial state.

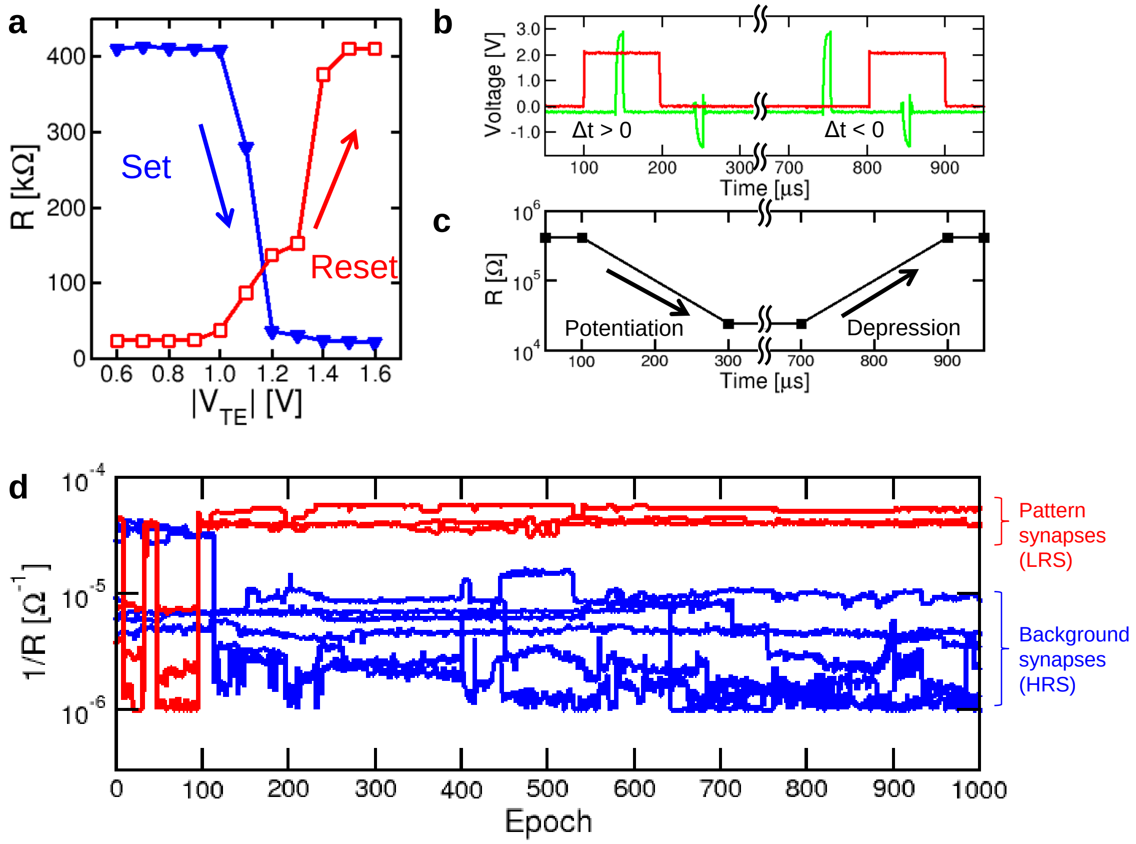

Figure S7 | High-speed STDP characteristics and learning. (a) Resistance R of a 1T1R RRAM synapse measured after the application of a pulse of width 10 μs, as a function of the absolute value $|V_{TE}|$ of the pulse voltage. In set experiments, the RRAM was prepared in the HRS and a positive voltage pulse was applied. In reset experiments, the RRAM was prepared in the LRS and a negative voltage pulse was applied. (b,c) Oscilloscope trace of overlapping PRE and POST spikes and corresponding R measured before/after each pair of spikes. Potentiation and depression occurs for $\Delta t > 0$ and $\Delta t < 0$, respectively, thus demonstrating STDP at the time scale of 10 μs TE spikes. (d) Synaptic weights 1/R as a function of time during a learning experiment in the neural network. Red and blue correspond to synaptic weights in the pattern and in the background, respectively. Pattern synapses tend to LRS while background synapses tend to HRS.

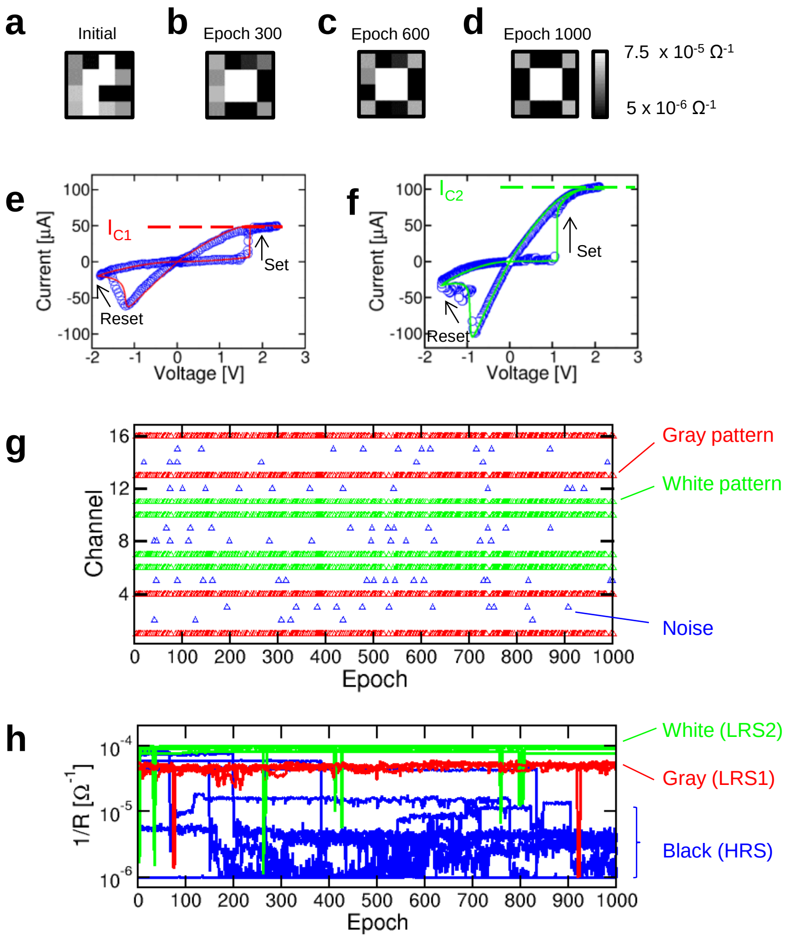

Figure S8 | Learning of a grayscale pattern. (a,b,c,d) Gray-tone code representation of the pattern weights at time 0, after 300 epochs, after 600 epochs, and after 1000 epochs. Light gray and dark gray indicate high and low conductance, respectively. The PRE input spikes had voltage amplitudes V_{G1} = 2.1 V and V_{G2} = 2.5 V, corresponding to compliance currents I_{C1} = 50 µA for synaptic level LRS1 and compliance currents I_{C2} = 100 µA for synaptic level LRS2, respectively, with LRS2 being more conductive than LRS1. (e,f) Measured I-V curves for a 1T1R synapse where the compliance current during set transition was I_{C1} and I_{C2}, respectively. As $I_{C2} > I_{C1}$, LRS2 shows a lower resistance than LRS1. (g) PRE spike sequence, indicating the presentation of the pattern (V_{G1} and V_{G2} pulses are shown in red and green, respectively) and the random noise (blue). (h) Synaptic weights 1/R as a function of time during a learning experiment in the neural network. Red and green correspond to synapses stimulated with V_{G1} and V_{G2}, respectively, while blue corresponds to the background. Pattern synapses tend to LRS1 (white) and LRS2 (gray), depending on the V_G, while background synapses (black) tend to HRS.

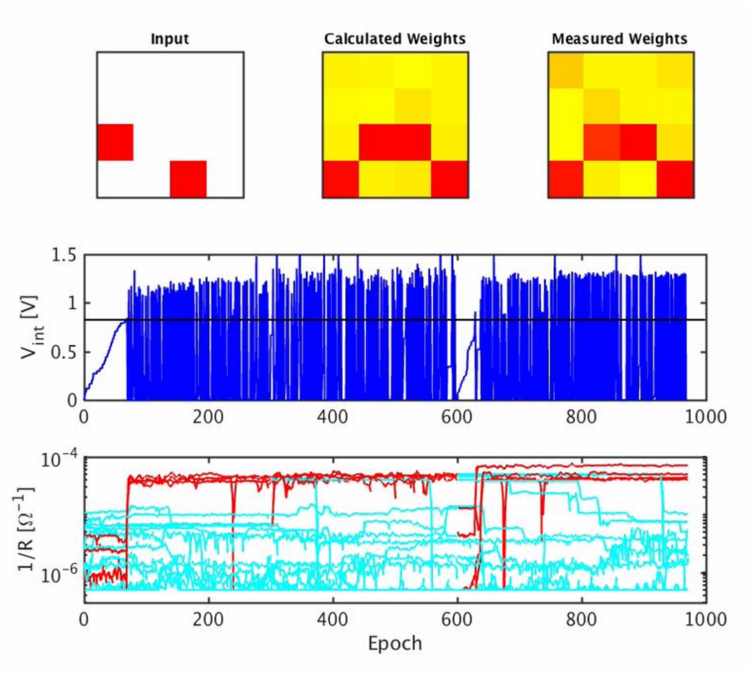

Supplementary Movie 1 | Static and dynamic learning within a 1-POST network. The movie shows the learning process for a 4x4 array of PRE fully connected to a single POST (same as Fig. 3). The color maps in the top row show the presented input (pattern or noise), the calculated weights according to a numerical simulation of the neural network, and the measured weights of the synaptic array. The figure in the mid row shows the internal potential V_{int} as a function of epochs, while the bottom row shows the synaptic weights 1/R as a function of epochs, for pattern synapses (red) and background synapses (light blue). The movie shows a total of 333 frames, (one every 3 epochs) at a representation speed of 30 frames per second (fps), resulting in representation time of 11 s, or 110% of the real time.

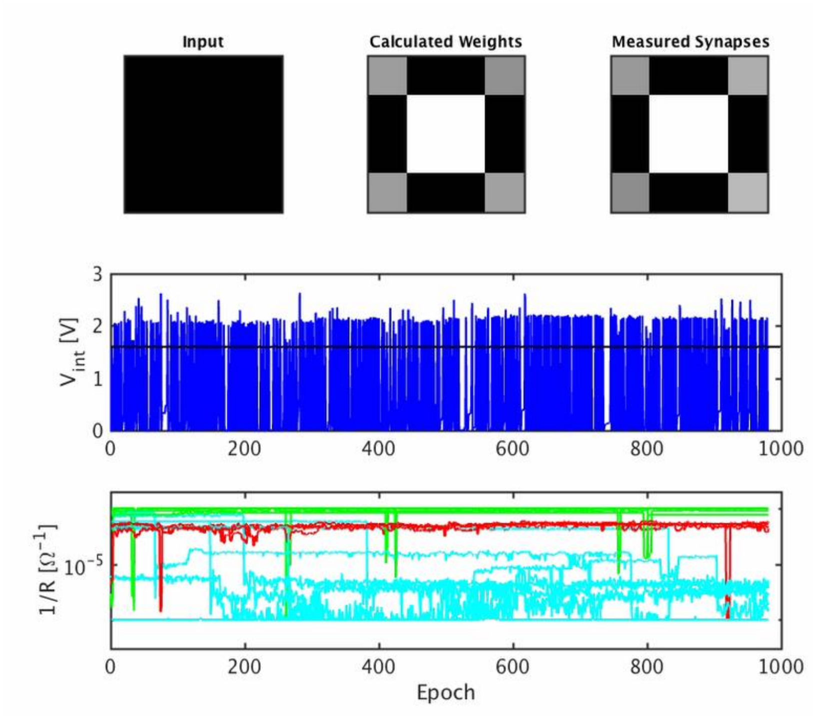

Supplementary Movie 2 | Learning of a grayscale pattern. The movie shows the learning process for a 4x4 array of PRE fully connected to a single POST with gray scale capability (same as Fig. S8). The color maps in the top row show the presented input (pattern or noise, light/dark gray represent high/low voltage), the calculated weights (light/dark gray represent high/low conductance) according to a numerical simulation of the neural network, and the measured weights of the synaptic array. The figure in the mid row shows the internal potential V_{int} as a function of epochs, while the bottom row shows the synaptic weights 1/R as a function of epochs, for white pixels (green), gray pixels (red), and background/black synapses (light blue). The movie shows a total of 333 frames, (one every 3 epochs) at a representation speed of 30 frames per second (fps), resulting in representation time of 11 s, or 110% of the real time.

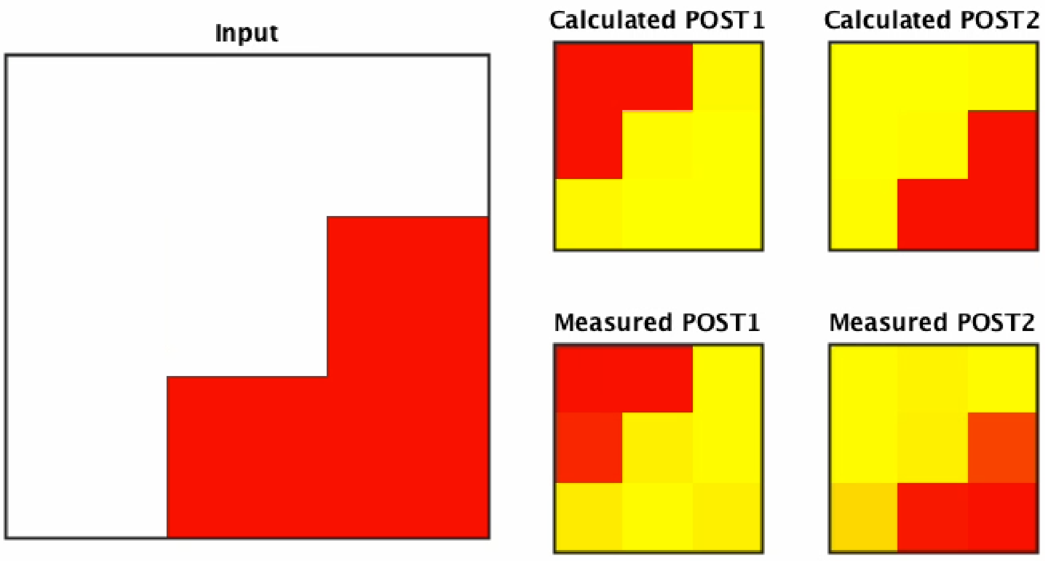

Supplementary Movie 3 | Static and dynamic learning within a 2-POST network. The movie shows the learning process for a 3x3 array of PRE fully connected to a 2 POSTs (same as Fig. 5). The big color maps on the left shows the presented input, which was either pattern 1, pattern 2, or noise. The 4 color maps on the right show the calculated weights of POST 1 and POST 2 (top row) and the measured weights of POST1 and POST2. In the first 1000 epochs, POST1 and POST2 specialize to pattern 1 (top bar) and pattern 2 (bottom bar), respectively. Then, both patterns are shifted by one pixel every 1000 epochs counter clockwise, and the synaptic weights are seen to track the movement of the patterns, demonstrating learning and tracking of dynamic patterns. The movie shows a total of 1000 frames, (one every 5 epochs) at a representation speed of 30 frames per second (fps), resulting in representation time of 33 s, or 67% of the real time.

REFERENCES

1. Calderoni, A., Sills, S., & Ramaswamy, N. Performance Comparison of O-based and Cu-based ReRAM for High-Density Applications. *Proc. Int. Memory Workshop* (IMW), 1-4 (2014). doi: 10.1109/IMW.2014.6849351
2. Ielmini, D. Modeling the universal set/reset characteristics of bipolar RRAM by field- and temperature-driven filament growth. *IEEE Trans. Electron Devices* **58**, 4309-4317 (2011). doi: 10.1109/TED.2011.21675132011
3. Ambrogio, S., et al. Analytical modeling of oxide-based bipolar resistive memories and complementary resistive switches. *IEEE Trans. Electron Devices* **61**, 2378–2386 (2014). doi: 10.1109/TED.2014.2325531.
4. Ambrogio, S., et al. Neuromorphic learning and recognition with one-transistor-one-resistor synapses and bistable metal oxide RRAM. *IEEE Trans. Electron Devices* **63**, 1508–1515 (2016). doi: 10.1109/TED.2016.2526647
